# The Uncommon ECG Signature of Hypokalemia: Left Main Coronary Equivalent

**DOI:** 10.1002/ccr3.71431

**Published:** 2025-11-09

**Authors:** Mahsa Borjzadehgashtaseb, Alireza Arzhangzadeh, Roozbeh Narimani Javid, Hamed Bazrafshan Drissi, Salma Nozhat, Sasan Shafiei, Davar Aledavood, Soorena Khorshidi

**Affiliations:** ^1^ Department of Cardiology, School of Medicine Shiraz University of Medical Sciences Shiraz Iran; ^2^ Research Center for Advanced Technologies in Cardiovascular Medicine, Cardiovascular Diseases Research Institute Tehran University of Medical Sciences Tehran Iran

**Keywords:** electrocardiogram, hypokalemia, ischemic heart disease, left main coronary equivalent

## Abstract

This case highlights that severe hypokalemia can mimic the ECG findings associated with left main coronary artery disease. Early recognition and appropriate correction of electrolyte disturbances are essential in managing such presentations and avoiding misdiagnosis and unnecessary invasive procedures.

## 1 Introduction

Hypokalemia is a frequently encountered electrolyte imbalance in clinical practice, often leading to a variety of electrocardiographic (ECG) changes. Common ECG findings in hypokalemia include flattened T waves, prominent U waves, and a prolonged QT interval, which can result in arrhythmias such as atrial fibrillation or ventricular tachycardia [1, 2]. In rare and severe cases, hypokalemia can manifest with more atypical and misleading ECG patterns, mimicking life‐threatening conditions like critical coronary artery stenosis [3]. One such presentation is the left main coronary ECG equivalent, a pattern typically associated with significant stenosis of the left main coronary artery [4]. Prompt recognition of this rare but serious manifestation of hypokalemia is essential to avoid unnecessary invasive procedures and prevent misdiagnosis, especially in patients without underlying coronary artery disease. This case report describes a rare instance of the left main coronary ECG equivalent in the context of severe hypokalemia, underscoring the importance of awareness regarding electrolyte‐related ECG alterations [5].

## 2 Case Presentation

A 38‐year‐old male with a history of opium inhalation, methadone use, and an 8‐pack‐year smoking history presented with recurrent episodes of nausea, non‐bloody, non‐bilious vomiting, constipation, and epigastric pain of 28 days' duration. The epigastric pain was constant, burning in nature, and exacerbated by food intake. The patient also reported unintentional weight loss, early satiety, and self‐induced vomiting by triggering the gag reflex due to abdominal discomfort. He had recently been prescribed omeprazole (one capsule daily). His past medical history included an appendectomy several years prior.

## 3 Investigations and Treatment

On physical examination, the patient appeared ill and cachectic. His vital signs were as follows: blood pressure 90/60 mmHg, heart rate 88 bpm, respiratory rate 16 breaths per minute, temperature 37.2°C, and oxygen saturation 95%. There was no palpable or visible lymphadenopathy. Abdominal examination revealed distension and tenderness localized to the epigastrium.

The patient had previously been evaluated at another hospital for similar symptoms. An upper gastrointestinal endoscopy performed at that time demonstrated grade B gastroesophageal reflux disease, multiple linear erosions in the distal esophagus, marked food retention in the stomach without gross lesions, and a chronic duodenal ulcer with severe pyloric stricture and bulb deformity. Passage of the endoscope into the second part of the duodenum (D2) was unsuccessful. He underwent three endoscopies over a 20‐day period at two different medical centers, with consistent findings. A diagnosis of gastric outlet obstruction was made based on these endoscopic findings.

**FIGURE 1:**
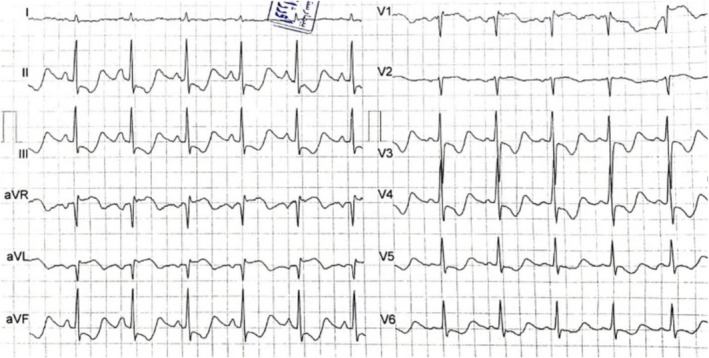
First ECG on admission, indicating wide spread ST‐segment depression and ST‐segment elevation in leads aVR, aVL, V1, and V2.

Upon admission to our hospital, the patient's ECG revealed diffuse ST‐segment depression, most prominent in the inferior leads and leads V3–V4, as well as ST‐segment elevation in leads augmented vector right (aVR), V1–V2, and augmented vector left (aVL), suggestive of a left main coronary artery pattern. The corrected QT interval was also prolonged (Figure 1).

A transthoracic echocardiogram demonstrated normal right and left ventricular ejection fractions and a left ventricular global longitudinal strain of −24.8%. No regional wall motion abnormalities or significant valvular heart disease were observed. Serial measurements of cardiac troponin were within the normal range, with no significant changes, ruling out acute coronary syndrome.

Laboratory investigations revealed the following values: white blood cell count of 9800/μL, hemoglobin of 12.8 g/dL, mean corpuscular volume of 67 fL, red blood cell count of 5.8 million/μL, red cell distribution width of 19.8%, platelet count of 384,000/μL, blood urea nitrogen of 32 mg/dL, creatinine of 1.4 mg/dL, sodium of 134 mEq/L, potassium of 2.5 mEq/L, calcium of 10.2 mg/dL, phosphorus of 4.4 mg/dL, magnesium of 2.1 mg/dL, albumin of 4.5 g/dL, and blood glucose of 148 mg/dL.

## 4 Outcome and Follow‐Up

**FIGURE 2:**
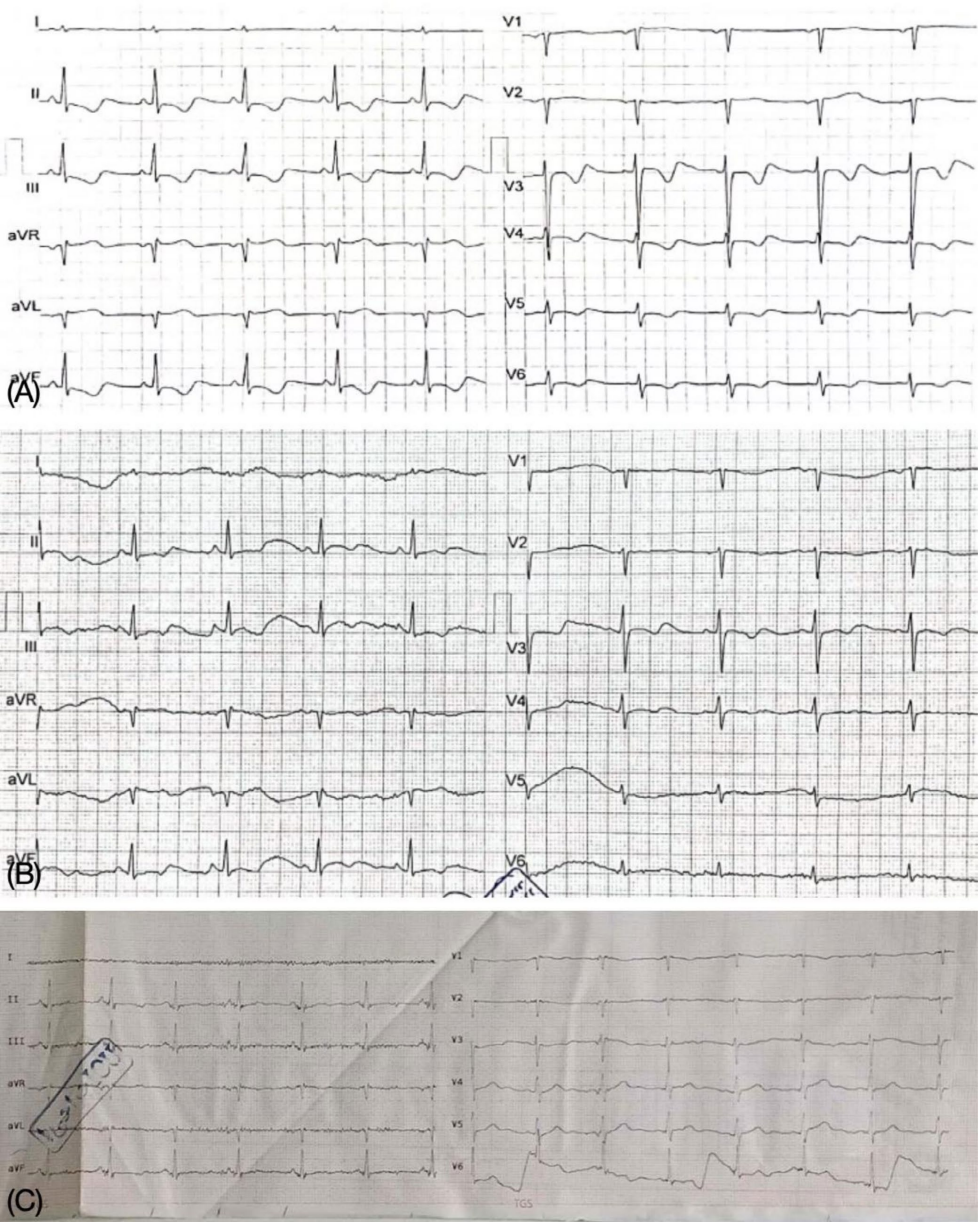
ECG post‐correction of hypokalemia on day 2 (A), on day 3 (B) after admission and at the time of discharge (C).

The patient was diagnosed with hypokalemia, with a serum potassium level of 2.5 mEq/L. Potassium levels were corrected via intravenous supplementation, reaching 4.0 mEq/L, and the ECG abnormalities subsequently resolved (Figure 2).

The patient was then transferred to the general surgery service for further management. An exploratory laparotomy confirmed the presence of a pyloroduodenal stricture causing gastric outlet obstruction. The patient underwent hemigastrectomy, gastrojejunostomy, and insertion of a Jackson‐Pratt drain. Postoperatively, the patient had an uneventful recovery and was discharged in stable clinical condition.

## 5 Discussion

Several case reports have explored the relationship between severe hypokalemia and the appearance of the left main coronary pattern on ECG, though such cases remain rare. For example, Chua CE, Choi E, and Khoo EYH described a 39‐year‐old female who presented with fever, diarrhea, vomiting, and atypical chest pain. Her initial ECG showed widespread, deep ST‐segment depressions and T‐wave inversions, with ST elevation in aVR. Despite these alarming ECG findings, her troponin levels were within normal limits, and echocardiography showed no abnormalities. A coronary CT angiogram revealed an Agatston score of zero, ruling out coronary artery disease. Her potassium level was 2.3 mmol/L, and after correction to 3.1 mmol/L, all ECG abnormalities resolved [6].

In our case, we were unable to perform a coronary CT angiogram due to the patient's critical condition and rising creatinine levels during hospitalization. However, the normal echocardiographic findings and absence of cardiac symptoms, coupled with normal troponin levels, strongly suggest that an ischemic cause of the left main pattern is unlikely.

Similarly, Mirijello et al. reported a 77‐year‐old female with hypokalemia and ECG abnormalities, including sinus rhythm and right bundle‐branch block with diffuse ST‐segment depression, most pronounced in leads V2–V3. These abnormalities resolved after potassium levels were corrected [7].

Humberto et al. reported an 84‐year‐old woman with cholecystitis, who presented with syncope. Her ECG showed atrial flutter with rapid ventricular response, ST elevation in aVR, and ST depression with T‐wave inversions in multiple leads. Despite normal echocardiography and troponin levels, hypokalemia secondary to antibiotic therapy was diagnosed, and her ECG abnormalities resolved following potassium replacement therapy [8]. As in our case, no additional workup such as coronary CT angiography was ordered, given the low probability of an ischemic cause.

Sethuraman et al. reported a case of an 84‐year‐old man with underlying triple vessel disease. He presented with generalized weakness and an ECG that suggested acute coronary syndrome due to ST‐segment depression in leads V4–V6 and first‐degree heart block. However, the patient exhibited no ischemic symptoms, and all ECG abnormalities resolved after potassium correction [9].

Petrov et al. described a 33‐year‐old male who presented with chest discomfort and upper abdominal pain, initially thought to be gastroenteritis. His ECG showed marked ST‐segment depression in leads II, III, aVF, and V1–V6. After potassium correction, the ECG returned to normal [10].

These reports, along with our case, highlight the critical importance of recognizing hypokalemia as a reversible cause of significant ECG changes, including patterns typically associated with left main coronary artery disease. Correct diagnosis and timely potassium replacement can prevent unnecessary invasive procedures and ensure proper management.

## 6 Conclusion

In conclusion, severe hypokalemia can mimic the left main coronary artery pattern on ECG, which is usually associated with critical coronary stenosis. Recognizing this reversible cause of alarming ECG findings is essential for clinicians, as it can be effectively managed through prompt potassium replacement. Awareness of this phenomenon can help avoid unnecessary diagnostic procedures, such as coronary angiography, in the absence of ischemic symptoms.

## Author Contributions

**Mahsa Borjzadehgashtaseb:** conceptualization, writing – original draft. **Alireza Arzhangzadeh:** supervision, writing – review and editing. **Roozbeh Narimani Javid:** investigation. **Hamed Bazrafshan Drissi:** project administration. **Sasan Shafiei:** methodology. **Davar Aledavood:** methodology. **Soorena Khorshidi:** conceptualization, writing – review and editing.

## Ethics Statement

Ethical approval is exempt/waived at our institution for this study.

## Consent

Written informed consent was obtained from the patient for publication of this case report and accompanying images. A copy of the written consent is available for review by the Editor‐in‐Chief of this journal on request.

## Conflicts of Interest

The authors declare no conflicts of interest.

## Data Availability

No data were used for the research described in the article.
